# *In Silico* Approach for Predicting Toxicity of Peptides and Proteins

**DOI:** 10.1371/journal.pone.0073957

**Published:** 2013-09-13

**Authors:** Sudheer Gupta, Pallavi Kapoor, Kumardeep Chaudhary, Ankur Gautam, Rahul Kumar, Open Source Drug Discovery Consortium, Gajendra P. S. Raghava

**Affiliations:** 1 Bioinformatics Centre, CSIR-Institute of Microbial Technology, Chandigarh, India; 2 Open Source Drug Discovery Consortium, Council of Scientific and Industrial Research (CSIR), Anusandhan Bhawan, New Delhi, India; UC Davis School of Medicine, United States of America

## Abstract

**Background:**

Over the past few decades, scientific research has been focused on developing peptide/protein-based therapies to treat various diseases. With the several advantages over small molecules, including high specificity, high penetration, ease of manufacturing, peptides have emerged as promising therapeutic molecules against many diseases. However, one of the bottlenecks in peptide/protein-based therapy is their toxicity. Therefore, in the present study, we developed *in silico* models for predicting toxicity of peptides and proteins.

**Description:**

We obtained toxic peptides having 35 or fewer residues from various databases for developing prediction models. Non-toxic or random peptides were obtained from SwissProt and TrEMBL. It was observed that certain residues like Cys, His, Asn, and Pro are abundant as well as preferred at various positions in toxic peptides. We developed models based on machine learning technique and quantitative matrix using various properties of peptides for predicting toxicity of peptides. The performance of dipeptide-based model in terms of accuracy was 94.50% with MCC 0.88. In addition, various motifs were extracted from the toxic peptides and this information was combined with dipeptide-based model for developing a hybrid model. In order to evaluate the over-optimization of the best model based on dipeptide composition, we evaluated its performance on independent datasets and achieved accuracy around 90%. Based on above study, a web server, ToxinPred has been developed, which would be helpful in predicting (i) toxicity or non-toxicity of peptides, (ii) minimum mutations in peptides for increasing or decreasing their toxicity, and (iii) toxic regions in proteins.

**Conclusion:**

ToxinPred is a unique *in silico* method of its kind, which will be useful in predicting toxicity of peptides/proteins. In addition, it will be useful in designing least toxic peptides and discovering toxic regions in proteins. We hope that the development of ToxinPred will provide momentum to peptide/protein-based drug discovery (http://crdd.osdd.net/raghava/toxinpred/).

## Introduction

The last decade has seen an unprecedented revival of interest in therapeutic peptides as potential drug candidates [1]. A plethora of research articles has been published every year demonstrating discovery of variety of novel therapeutic peptides *e.g.* tumor homing peptides [2], cell penetrating peptides [3], anti-microbial peptides [4], anticancer peptides [5], [6], [7], *etc.* and applications of these peptides in various diseases like cancer, diabetes, cardiovascular diseases, *etc.* As a result of these efforts, rate of therapeutic peptides entering into clinical trials has improved significantly over the decade [1]. However, despite the discovery of hundreds of such therapeutic peptides, only few peptide-based drugs have made it to the market.

Peptides have numerous advantages over small molecules that include high biological activity, high specificity, low production cost, and high penetration [1], [5]. However, toxicity, immunogenicity and stability remain the main concerns in the development of peptide-based drugs. Stability of peptides can be enhanced by various ways [8], including incorporation of D-amino acids (making peptides protease resistant), changing the backbone chemistry, cyclization, and incorporation of α-aminoxy amino acids [9]. Similarly, there are numerous *in silico* tools, which can predict the immunogenicity of the peptides [10], [11], [12], [13], [14], but there is hardly any way/method to predict the toxicity of peptides. Computational methods for predicting toxicity of peptides not only save time and money, but also facilitate the designing of better therapeutic peptides with low toxicity while retaining the functionalities.

Keeping these facts in mind, in this study, for the first time, an attempt has been made to develop an *in silico* method for predicting toxicity of peptides. Toxic peptides have been collected from various databases/studies including ATDB [15], DBETH [16], BTXpred [17], NTXpred [18], Arachno-Server [19], Conoserver [20]. *In silico* models have been developed using the machine-learning technique support vector machine (SVM), for discriminating toxic peptides from non-toxic peptides. In addition, various motifs from toxic peptides were discovered and used for toxicity prediction.

## Materials and Methods

### Dataset Creation

**Figure 1:**
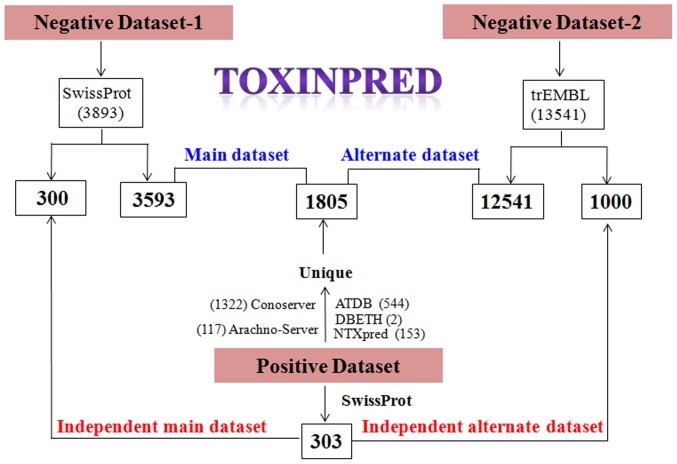
Overview of datasets’ creation.

We extracted small toxins (proteins/peptides) from different databases and studies that include ATDB [15], Arachno-Server [19], Conoserver [20], DBETH [16], BTXpred [17], NTXpred [18], and SwissProt [21]. We removed all proteins/peptides having more than 35 residues or any non-natural amino acid. As a result, 1805 unique toxic proteins/peptides were obtained. By employing the similar criteria, toxic proteins/peptides were also searched in SwissProt database using keyword KW800 (keyword 800 stands for toxin as molecular functions). A total of 803 toxic proteins, having length less than 35 amino acids were obtained. It is possible that many toxic peptides obtained from various databases could also be present in SwissProt. Therefore, identical toxic proteins/peptides were removed and finally we got 303 unique toxic proteins/peptides from SwissProt. These proteins/peptides were considered as toxic peptides or positive examples. Though it is possible to extract well-annotated or experimentally validated toxic peptides, but it is difficult to obtained non-toxic peptides. Therefore, to create a negative dataset, we have searched protein/peptide sequences in UniProt using keywords NOT KW800 NOT KW20 (keyword 800 and 20 stand for toxin and allergen as molecular functions). Proteins/peptide sequences having length less than 35 amino acids were extracted. After removing sequences with non-natural amino acids, two types of negative datasets were created; first dataset consists of 3893 sequences from SwissProt (NOT KW800 NOT KW20) and second dataset consists of 13541 sequences from TrEMBL (keyword NOT KW800 AND KW33090) [21]. While searching non-toxins in TrEMBL, additional keyword plant proteins were applied as search criteria as most of the plants are edible and therefore, the probability of plant proteins/peptides to be toxic is very low. Above toxic and non-toxic peptides/proteins were used to generate various datasets for training, testing and evaluating our models developed for predicting toxicity of peptides (Figure 1). Following is the brief description of these datasets:

#### Main and alternative datasets

The main dataset used for training and testing of SVM models was generated from experimentally validated toxic peptides (obtained from various databases) and well-annotated non-toxin peptides/proteins obtained from SwissProt. It includes 1805 toxic peptides as positive examples and 3593 non-toxic peptides as negative examples. In addition, we also generated an alternate dataset, which is similar to the main dataset except negative examples. It consists of 1805 toxic-peptides/proteins as positive examples and 12541 non-toxin peptides/proteins obtained from TrEMBL (instead of SwissProt).

#### Independent datasets

In order to evaluate biases in the performance of developed models, we created different independent datasets. First independent dataset comprises of 303 toxic proteins/peptides (called positive examples) and 300 non-toxic peptides/proteins or negative examples extracted from SwissProt. None of the negative or positive examples was included in the main dataset. This dataset is referred as main independent dataset and used for evaluating models developed on the main dataset. In order to evaluate the performance of models developed on alternate dataset, we developed another independent dataset referred as alternate independent dataset. This dataset consists of 303 positive examples extracted from SwissProt and 1000 negative examples extracted from TrEMBL, which were not included in the alternate dataset.

### Models Developed using Support Vector Machine

There are a number of machine learning techniques used for building prediction models. In this study, support vector machine (SVM) has been used for building models [22]. We developed SVM based models using a freely available software SVM^light^(version 6.02).

### Amino Acid Composition

The amino acid composition is defined as the fraction of each amino acid in a peptide and it can be calculated by the following equation:
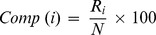



Where Comp (*i*) is the percent composition of amino acid (*i*); *R_i_* is the numbers of residues of type *i*, and *N* is the total number of residues in the peptide.

### Dipeptide Composition

The dipeptide composition is advantageous over simple amino acid composition as it provides a composition of a pair of residues (*e.g.* Gly-Gly, Gly-Leu, *etc.*) present in the peptide. Dipeptide composition can be calculated using the following equation:
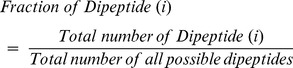



Where dipeptide (*i*) is one out of 400 dipeptides.

### Binary Profile of Patterns

The binary profile is a commonly used feature for developing prediction models, and it has been used previously in many methods [23], [24]. The binary profile encapsulates information of both composition as well as order of amino acids in peptides. Binary profiles of N- and C-termini of peptides were generated as described previously [25].

### Two Sample Logos

Two sample logos were generated using online two-sample logo software [26]. The sequence logo provides the position specific preference of amino acids in peptides.

### Motif Identification

We used Multiple Em for Motif Elicitation (MEME, version 4.9.0) program [27] for the identification of prominent motifs in the toxic peptides. We have identified a number of motifs, and these motifs were used further to assign unknown sequences as toxic or non-toxic by scanning of these motifs. For this purpose, we have used another application of MEME suite called Motif Alignment & Search Tool (MAST). E-value is very crucial in the MAST output, so efficacy of this method was calculated at different E-values (10–10^−7^).

### Hybrid Approach

Hybrid approach combines the motif information with SVM output for biologically more reliable prediction of toxic peptides. In this approach, first, various motifs are searched in the query peptides, and if any of the motifs of toxic peptide is present, it’s SVM score is increased by the value of 5. This final score is used for the prediction, which in this case, will always be predicted as toxic peptide irrespective of SVM prediction. In this way, hybrid approach adds an extra advantage in the prediction.

### Quantitative Matrix

QMs have been used successfully in the past to predict MHC binders [28] and TAP binders [29]. In this study, we have also generated QMs for both datasets (main and alternate datasets). QM was generated on the basis of relative frequency of each amino acid at every position (ranging from position 1 to position 35). This matrix represents the contribution of each residue (A to Y) at every position (from 1 to 35), resulting into a matrix of dimension 20×35. Rows represent the residues and columns represent the position information. QMs were generated for positive and negative peptides for both datasets (main and alternate dataset). Resultant matrix was obtained by subtracting negative QM from the positive QM in each dataset. Then, this resultant QM was used for further analysis as this matrix represents the relative contribution of each residue at every position relatively in positive and negative peptides, in each dataset. In a similar way, QM was generated for dipeptide motifs where each row represents the dipeptide and each column represents the position of that dipeptide, resulting into a matrix of dimension 400×34 (this information has been provided at “Matrices” module of ToxinPred webserver). Query peptides were scanned on this resultant QM to get the resultant score (cumulative score) and to see what is the frequency of every residue of query peptide at the corresponding position.

### Evaluating the Performance of Models

We have evaluated the performance of different models using cross validation techniques, which involved random division of dataset into *n* number of sets. The training and testing were carried out for *n* times, each time one set was used for testing and remaining (*n*-1) sets were used for training. We used five (*n = *5) and ten (*n = *10) fold cross validation techniques. Various standard parameters like Sensitivity, Specificity, Accuracy, and Matthew’s correlation coefficient (MCC) were used for assessing the performance of models. These parameters can be computed as described in previous studies [25], [30]. In addition, to validate the SVM model, the performance of SVM models were also evaluated on independent datasets.

## Results

### Amino Acid Composition Analysis

**Figure 2:**
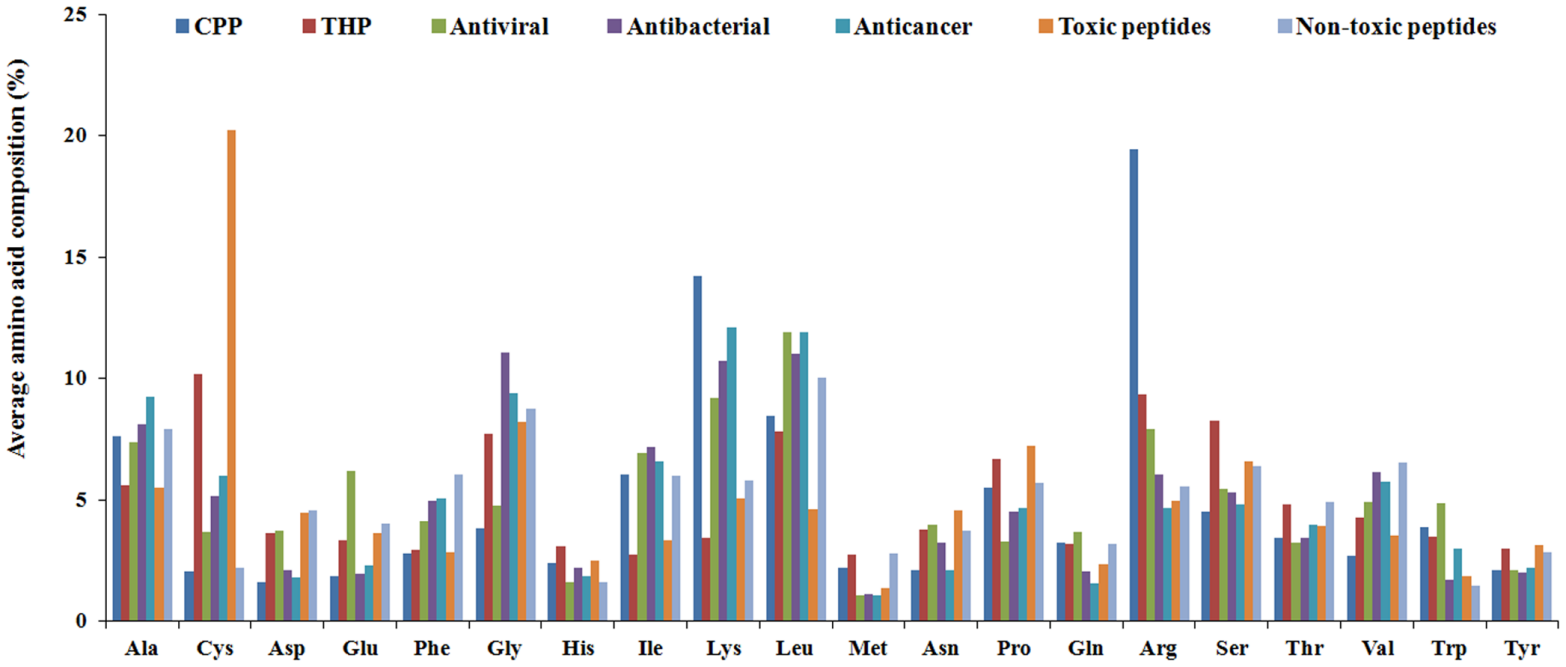
Comparison of average amino acid composition between various classes of therapeutic peptides.

In order to understand the nature of toxic peptides, first, percent average amino acid composition of toxic peptides and non-toxic peptides were calculated and compared. The result of this analysis is shown in Figure 2. As demonstrated, certain residues like Cys, His, Asn, and Pro were found to be dominated in toxic peptides compared to non-toxic peptides. On the other hand, Val, Thr, Arg, Gln, Met, Leu, Lys, Ile, Phe, and Ala were dominant in non-toxic peptides. Next, we wanted to know how toxic peptides are different from the other classes of therapeutic peptides, therefore to address this, percent average amino acid composition of various other classes of therapeutic peptides (cell penetrating peptides, tumor homing peptides, anti-viral peptides, anti-bacterial peptides, anti-cancer peptides) were calculated and compared with toxic peptides. The result of this comparison is shown in Figure 2. It was observed that the composition of Cys was exceptionally high, and composition of Asn and Pro was slightly higher in toxic peptides in comparison to other peptides. Similarly, composition of Arg, Leu, Lys, and Ile in toxic peptides was significantly low as compared to other classes.

**Figure 3:**
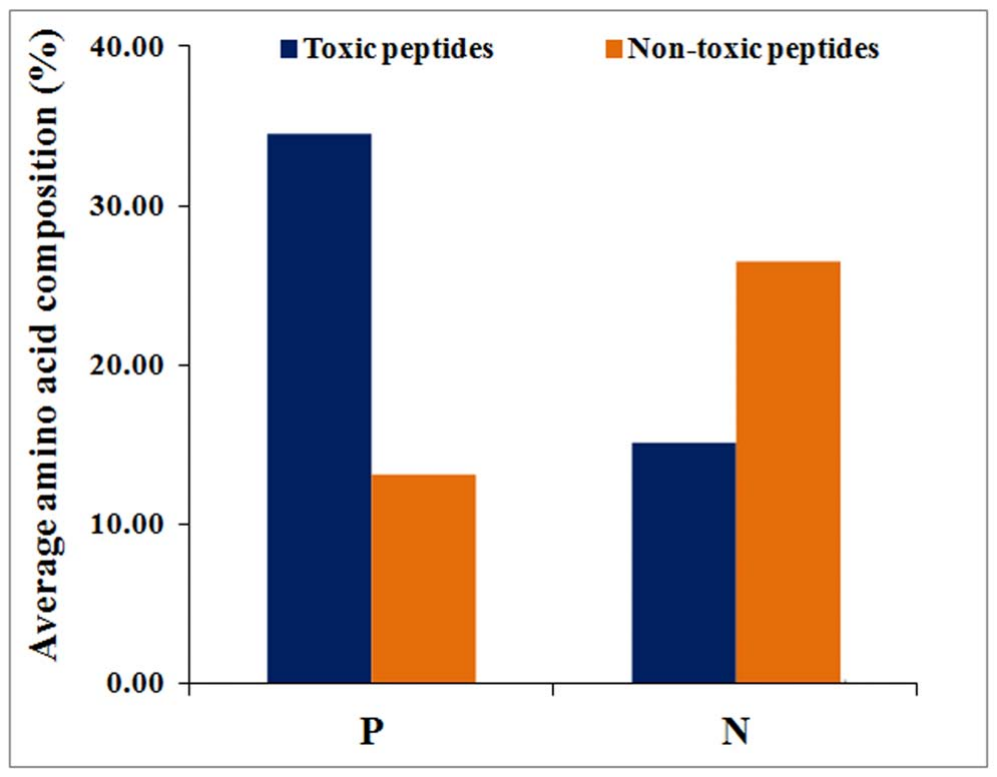
Comparison of average amino acid composition of preferred residues between toxic and non-toxic peptides.

In addition, we also looked at the cumulative difference between the average composition of residues dominating in both toxic and non-toxic peptides (Figure 3). We categorized the significant residues (p<0.05) in two groups: (i) positive dominating (Cys, His, Asn and Pro), which are abundant in toxic peptides, and (ii) negative dominating (Ala, Phe, Ile and Val), which are abundant in non-toxic peptides. As shown in Figure 3, there is a significant difference in the average composition of positive and negative dominant residues between toxic and non-toxic peptides.

### Residue Preference

**Figure 4:**
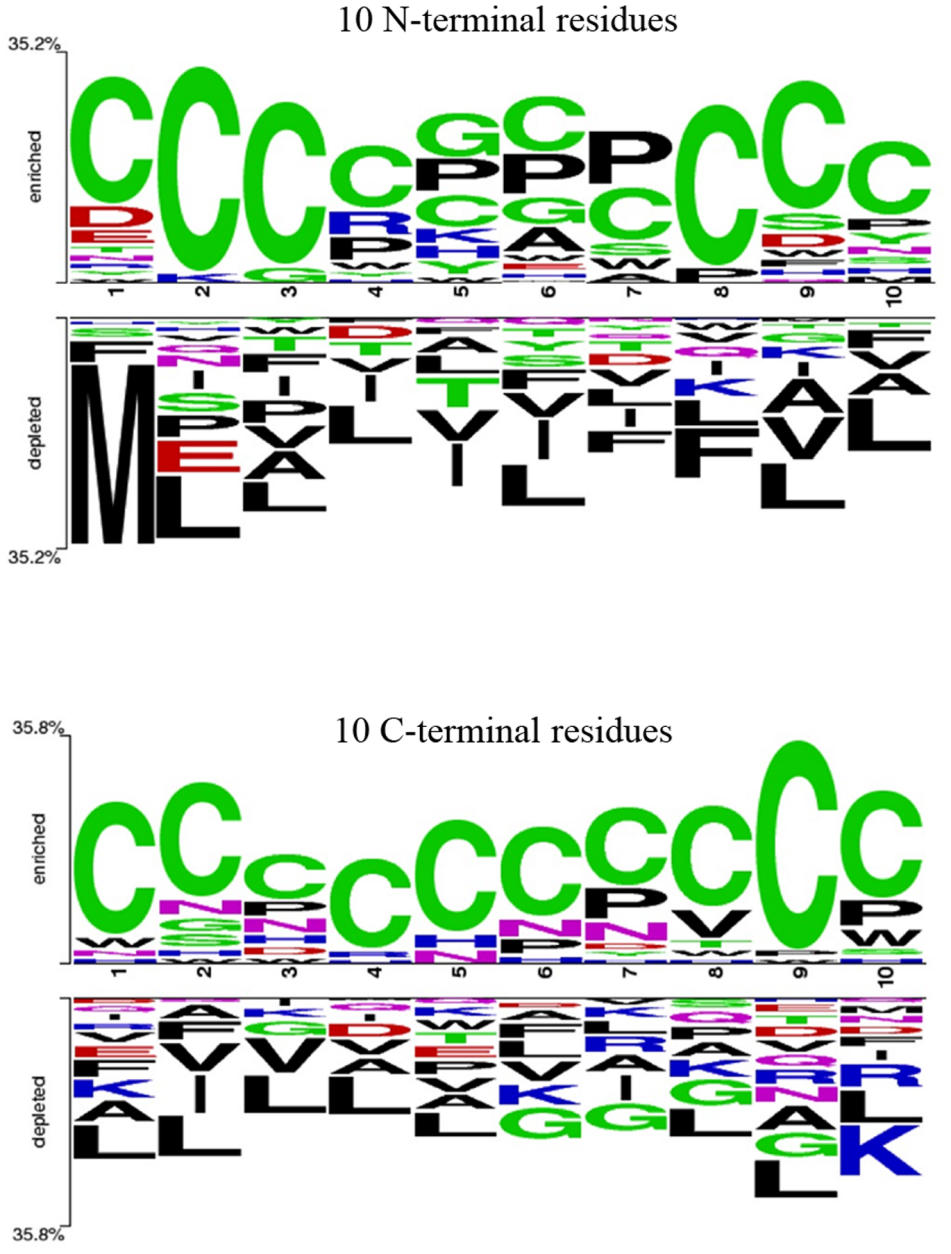
Sequence logos of (A) first ten residues of N-terminus and (B) last ten residues of C-terminus of toxic peptides, where size of residue is proportional to its propensity (main dataset).

Next, we wanted to analyze the residue preference at various positions at both the termini in toxic and non-toxic peptides. Therefore, two-sample logos were made from main dataset using the two-sample logo software [26]. The results of two-sample logos of N- and C-terminal of main dataset and alternate dataset were shown in Figure 4 and Figure S1 in file S1 respectively. Similar to amino acid composition, Cys was preferred at almost all positions at both C- and N-terminal in toxic peptides. In addition, Pro, Gly, Arg and Ser were also found to be preferred at few positions at N-terminus while Val, Asn and His were preferred at few positions at C-terminus in toxic peptides. In contrast, Met, Leu, Phe, and Ile were preferred at various positions at N-terminus, while Leu, Gly, and Lys were preferred at various positions at C-terminus of non-toxic peptides. Overall Leu was found to be dominant in non-toxic peptides at most of the positions at both N- and C-terminal of peptides.

### Amino Acid Composition-based SVM Model

Preliminary composition analysis has revealed that certain types of residues are abundant in toxic peptides. Thus, it is possible to discriminate toxic peptides from non-toxic peptides based on amino acid composition. Therefore, we developed an SVM model using amino acid composition as input feature. Composition based SVM model achieved maximum accuracy of 93.92% with MCC and AUC values 0.87 and 0.97, respectively on main dataset (Table 1). Similarly, composition based SVM model on alternative dataset achieved accuracy of 96.70% with MCC and AUC values 0.86 and 0.99 respectively (Table S1 in file S1). In addition models were build using spilt amino acid composition, there performance has are summarized in Table 1 and Table S1 in file S1. The models based on split amino acid composition (C5AAC, C10AAC, N10AAC, and N10AAC) did not perform better than the whole composition-based models (Table 1 and Table S1 in file S1). Model based on composition of 10 C-terminal amino acid (C10AAC, main dataset) achieved maximum accuracy of 90.69% with MCC and AUC values 0.80 and 0.94 respectively.

**Table 1:** The performance of SVM-based models developed on main dataset using various types of composition like residue, dipeptide, terminal residues composition.

Features	Parameters	Threshold	Sensitivity	Specificity	Accuracy	MCC	AUC
**AAC**	t:2 g:0.005 c:5 j:1	−0.4	92.91	94.43	93.92	0.87	0.97
**C5AAC**	t:2 g:0.001 c:0.5 j:3	−0.3	83.74	83.67	83.69	0.65	0.88
**C10AAC**	t:2 g:0.005 c:10 j:3	−0.3	88.66	91.73	90.69	0.80	0.94
**N5AAC**	t:2 g:0.001 c:0.5 j:4	−0.1	81.76	81.47	81.55	0.59	0.88
**N10AAC**	t:2 g:0.005 c:1 j:3	−0.4	90.41	86.94	88.11	0.75	0.94
**DPC**	t:2 g:0.001 c:5 j:1	−0.4	93.80	94.85	94.50	0.88	0.98

AAC, amino acid composition; DPC, dipeptide composition; C5AAC, amino acid composition of last five C-terminal residues; C10AAC, amino acid composition of last ten C-terminal residues; N5AAC, amino acid composition of first five N-terminal residues; N10AAC, amino acid composition of first ten N-terminal residues; MCC, Matthew’s correlation coefficient; AUC, area under the curve.

### Models Based on Dipeptide Composition

Dipeptide composition is considered better feature as compared to amino acid composition as dipeptide composition encapsulates the information of both amino acid fraction and the local order of amino acids. Thus, we also developed SVM models based on dipeptide composition. The performance of dipeptide composition-based model was better than whole composition-based model developed on both main and alternate datasets (Table 1 and Table S1in file S1). Dipeptide composition-based model developed on main dataset achieved maximum accuracy of 94.50% with MCC and AUC values of 0.88 and 0.98 respectively (Table 1). Similarly, on alternate dataset accuracy was 98.64% with MCC 0.94 (Table S1 in file S1).

### Binary Profile-based SVM Model

Since it was found that certain residues, including Cys, Pro, Arg, Ser, Val, Asn and Gly are preferred at various positions at both the termini of the peptides, binary profiles were generated to incorporate amino acid order information in the model. We have used the following three approaches:

#### N-terminal approach

In this approach, we have generated binary profiles of dimension 5×20 and 10×20 respectively by extracting 5 and 10 N-terminus residues from each peptide in both the datasets. These profiles were then used as input features to develop SVM model. Comparison of the performances of binary-based SVM models developed on main dataset is shown in Table 2. The performance of the model developed on NT10 dataset was better than that of the model developed on NT5 dataset. NT10 based model achieved maximum accuracy of 91.63% with MCC and AUC values 0.82 and 0.96 respectively (Table 2). However, this trend is reversed for models developed on alternate dataset. For alternate dataset, NT10 based models performed poorer than NT5 based model (Table S2 in file S1).

**Table 2:** The performance of binary profile-based models developed on main dataset.

Feature	Parameters	Threshold	Sensitivity	Specificity	Accuracy	MCC	AUC
CT5	t:2 g:0.5 c:1 j:1	−0.5	84.67	86.8	86.09	0.70	0.90
CT10	t:2 g:0.1 c:5 j:1	−0.3	91.50	91.81	91.70	0.82	0.96
NT5	t:2 g:0.5 c:5 j:2	−0.4	84.32	87.79	86.78	0.70	0.91
NT10	t:2 g:0.1 c:5 j:5	−0.3	91.13	91.89	91.63	0.82	0.96

MCC, Matthew’s correlation coefficient; AUC, area under the curve.

#### C-terminal approach

Similar to N-terminal approach, models based on binary profiles were developed using CT5 and CT10 datasets. For main dataset, the performance of CT5 and CT10 based models was almost similar to N-terminal approach (Table 2). The CT10 based model performed better than CT5 based model. However, for alternate dataset, both models (CT5 and CT10) performed with almost equal accuracy (Table S2 in file S1).

### Motif-based Prediction

Motif information has been used previously in many studies to classify two different classes of peptides. We also applied the similar strategy and found out 20 prominent motifs present in the toxic peptides at E-value of 10. This motif information was used further in MAST for the prediction of toxic peptides at different E-value ranging from 10 to 10^−7^. Probability of correct predictions (PCP) was 93.40% at E-value of 10^−7^ as shown in Table 3. Similar results were obtained for alternate dataset (Table S3 in file S1). Although, PCP was satisfying, but percent coverage is bit less, so we integrated this method with dipeptide composition-based SVM method to improve the performance of the model.

**Table 3:** The performance of motif-based model developed on main dataset.

E-value	PCP	%Coverage
**10**	40.56	93.54
**1**	48.27	90.08
**0.1**	59.11	86.28
**0.01**	69.31	82.31
**1E-02**	78.07	78.29
**1E-04**	85.16	74.83
**1E-05**	89.15	71.77
**1E-06**	92.12	68.25
**1E-07**	93.40	64.17

PCP; probability of correct prediction.

### Hybrid Prediction Model

Since MEME/MAST method is very efficient in predicting toxic peptides, but with very little coverage (Table 3 and Table S3 in file S1). Therefore, we have developed a hybrid method by combining MEME/MAST method with the dipeptide-based SVM model in order to improve the performance of the model. The performance of hybrid model is shown in Table 4. Hybrid model achieved maximum accuracy (at E-value 10) of 98.41% with MCC and AUC values 0.96 and 0.99 respectively (Table 4). Hybrid model developed on alternate dataset performed similar to the model developed on main dataset (Table S4 in file S1).

**Table 4:** The performance of model developed using motifs and dipeptide composition on main dataset.

E-value	Sensitivity	Specificity	Accuracy	MCC	AUC
**10**	99.39	97.91	98.41	0.96	0.99
**1**	98.89	97.91	98.24	0.96	0.99
**0.1**	98.39	97.91	98.07	0.96	0.99
**0.01**	97.78	97.91	97.87	0.95	0.99
**0.001**	97.17	97.91	97.67	0.95	0.99
**0.0001**	96.84	97.91	97.55	0.95	0.99
**0.00001**	96.62	97.91	97.48	0.94	0.99
**0.000001**	96.29	97.91	97.37	0.94	0.99
**0.0000001**	95.84	97.91	97.22	0.94	0.99

MCC, Matthew’s correlation coefficient; AUC, area under the curve.

### Quantitative Matrix–based Prediction of Toxic Peptides

**Figure 5:**
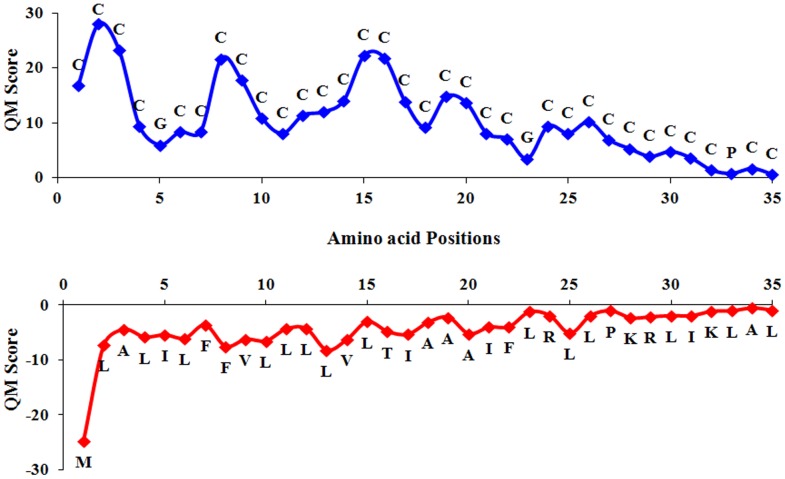
Maximum and minimum scoring residues at every position as observed in quantitative matrix (main dataset).

The quantitative matrices were used for prediction of toxic peptides. From five fold cross validation, accuracy values 88.00%, 95.81%, 89.65% and 95.78% were achieved for monopeptide main dataset (Single_main), monopeptide alternate dataset (Single_alternate), dipeptide main dataset (Dipep_main), dipeptide alternate dataset (Dipep_alternate) matrices, respectively and the corresponding MCC values were 0.73, 0.81, 0.77, 0.81, respectively (Table 5). Though the performance of the QM–based method was poorer than that of the SVM based model, but it has more biological significance as it gives information about each amino acid contribution at all positions. Also to get more insights into the relative contribution of residues at each position (1 to 35), graphical representation (Figure 5 and Figure S2 in file S1) demonstrated the highest and lowest scoring residues at every position in single and dipeptide matrices for main and alternate datasets, respectively. Here, the minimum and maximum scoring residues have been used to show the range of scores at every position.

**Table 5:** The performance of quantitative matix based method on various datasets.

Matrix	Threshold	Sensitivity	Specificity	Accuracy	MCC	AUC
**Single_main**	20	80.46	92.09	88.00	0.73	0.92
**Single_alternate**	20	75.43	98.98	95.81	0.81	0.97
**Dipep_main**	5	74.10	98.07	89.65	0.77	0.95
**Dipep_alternate**	5	73.29	99.28	95.78	0.81	0.98

MCC, Matthew’s correlation coefficient; AUC, area under the curve.

### ROC Plots

**Figure 6:**
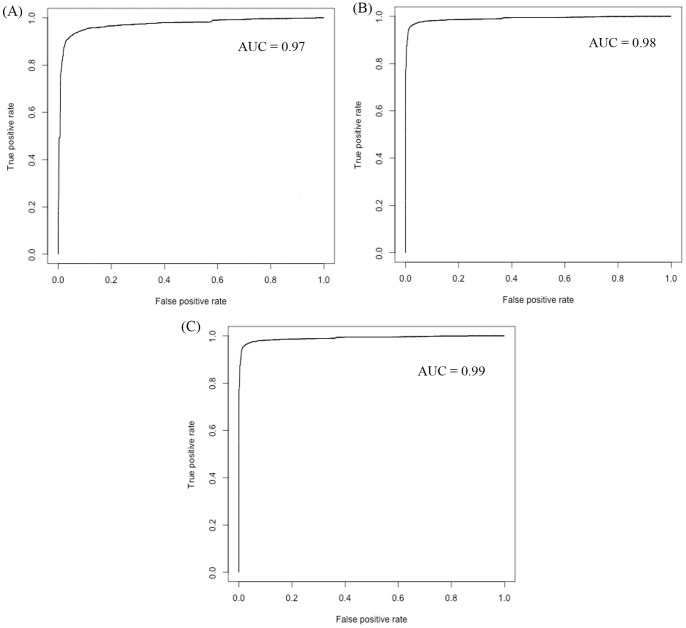
ROC curves of support vector machine models based on (A) amino acid composition, (B), dipeptide composition, and (C) hybrid approach.

ROC curves were generated for threshold-independent evaluation of our models. ROC curves with area under the curves (AUC) were generated using ROCR statistical package. As shown in Figure 6, hybrid method performed better than the whole composition and dipeptide composition based methods (Figure 6).

### Performance on Independent Datasets

It has been shown in the past that there are chances of over-optimization in cross-validation techniques. In order to evaluate the unbiased performance of dipeptide-based model, we tested the performance on main independent dataset and achieved accuracy of 75.79% with MCC 0.52. Similarly, on alternate independent dataset, model achieved accuracy of 87.87% with MCC value 0.67. Surprisingly, the performance of models decreased significantly on independent datasets. It might be due to two reasons, either model got over-optimized and achieved high performance during cross-validation or it might be due to the fact that independent datasets are different from the training datasets. Our independent datasets consist of toxic proteins/peptides extracted from SwissProt, and it is possible that these proteins/peptides have different properties.

In order to address the above problem, we created independent datasets using an alternate strategy. We first, mixed toxic peptides obtained from SwissProt and other databases and then randomly extracted 303 peptides for developing an independent dataset. The remaining toxic peptides were used for building main and alternate datasets. We again trained and tested our dipeptide-based models on modified main and alternate datasets. The performances of these models on modified datasets were nearly the same. We tested dipeptide-based model on the new main independent dataset and achieved accuracy 90.55 with MCC 0.81. Similarly, we evaluated the performance of dipeptide-based model on new alternate independent dataset and achieved accuracy 96.01 with MCC 0.89. These results suggest that performance of dipeptide-based model is reasonably good on independent datasets and nature of toxic peptides obtained from SwissProt and other databases is different.

### Implementation and Utility of ToxinPred

**Figure 7:**
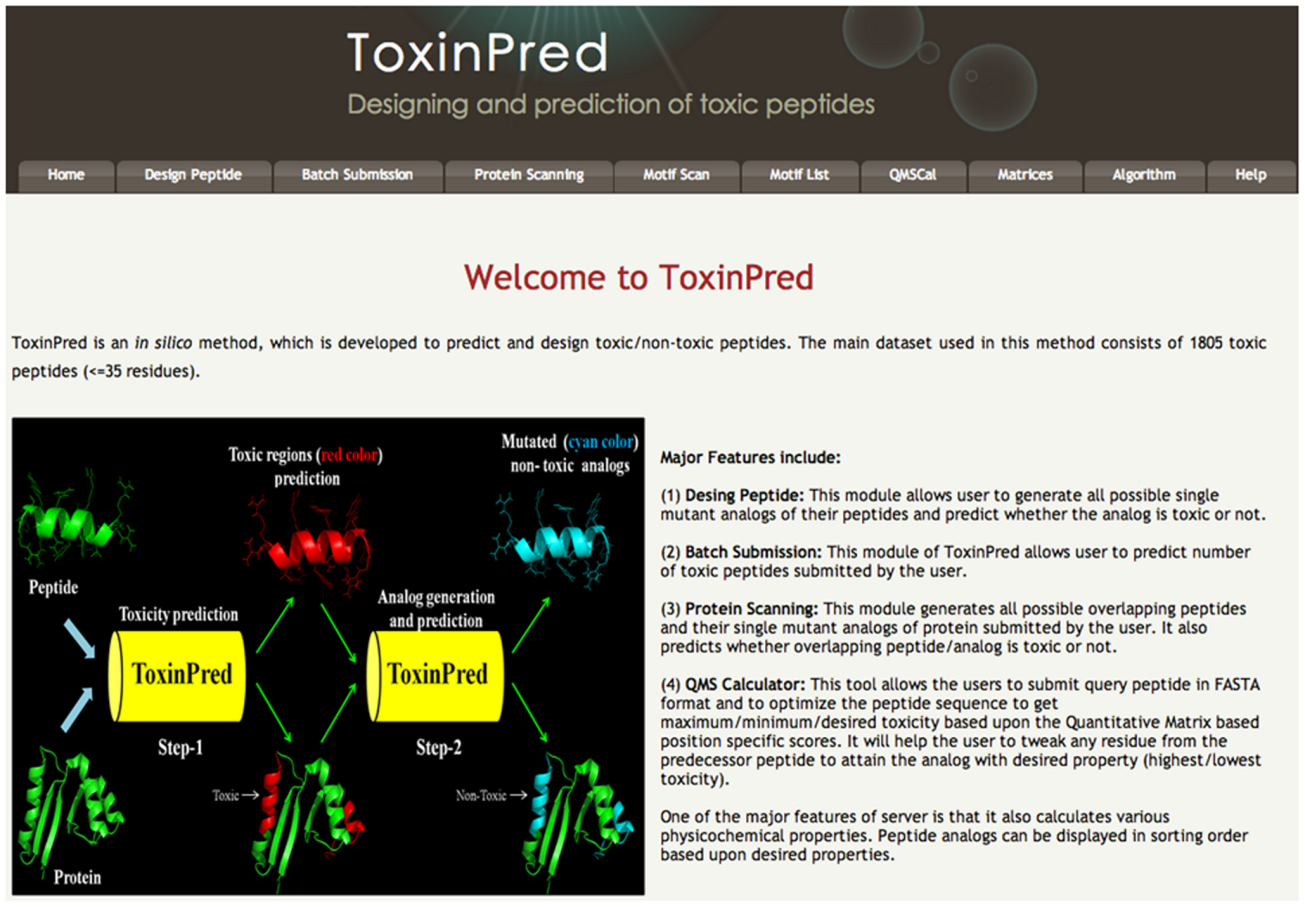
Schematic representation of ToxinPred webserver.

In order to serve the scientific community, we have implemented our best models (dipeptide and hybrid) in a user-friendly web server ‘ToxinPred’ (Figure 7). ToxinPred provides the facility for both the prediction and designing of toxic and non-toxic peptides. The description of various tools is as follows:

#### (1) Design peptide

This server allows users to design peptides of desired toxicity. In this module, server first generates mutant peptides from submitted peptide using all possible single mutations, secondly it predict toxicity (SVM score proportional to toxicity) for each mutants. Finally, user can select the best mutant peptide and may submit the best mutant for generating its mutant with their toxicity score. This module will assist users in designing and discovering peptides of desire toxicity.

#### (2) Batch submission

One of the challenges in the field of drug discovery is virtual screening of drugs. This module allows users to perform virtual screening of peptides for discovering the best peptide with desired toxicity. One may submit multiple peptides to ToxinPred server, and it will predict toxicity of each peptide.

#### (3) Protein scanning

This module is useful to identify toxic regions in a protein sequence. This module first generates all possible overlapping peptides and subsequently, server predicts toxicity of each overlapping peptides. Thus, user can easily identify highly toxic regions in a protein. User can obtain results in both tabular and graphical format. User may remove or alter the protein sequence in the required manner with minimum mutations in order to reduce/enhance the toxicity of their peptides/proteins.

#### (4) QMS calculator

This tool allows users to optimize the peptide to get maximum/minimum/desired toxicity based upon the QM-based position specific scores. It will help the users to tweak any residue from the predecessor peptide to attain the analog with desired property (highest/lowest toxicity). User can identify residues that may reduce/enhance the toxicity drastically using our server.

#### (5) Motif scanning

We discovered a list of motifs from toxic peptides using MEME/MAST software. These motifs are available on ToxinPred and server allows users to search these motifs in their protein sequences. In simple words, our server allows users to scan/discover toxic motifs in their proteins or peptides.

## Discussion

The aim of the present study is to develop an *in silico* method to predict toxicity of therapeutic peptides/proteins. The prediction of toxicity of therapeutic peptides/proteins before their synthesis is very important for saving both time and money consumed in developing peptide/protein-based drugs. Our main dataset contain 1805 known toxic peptides collected from various databases and 3893 non-toxic peptides collected from SwissProt. Since SwissProt has well annotated entries, these peptides could be true representatives of non-toxic peptides. Alternative dataset was generated in this study for creating realistic conditions where non-toxic peptides are many fold more than toxic peptides. Thus in alternate dataset, we have used 13,541 non-toxic peptides obtained from TrEMBL database.

Preliminary analysis of toxic peptides (by calculating percent amino acid composition and two sample logos) revealed that Cys is present in a high proportion as well as preferred at almost all positions in toxic peptides. In addition, composition of Pro, Asp and His was found to be higher in toxic peptides in comparison to non-toxic peptides. This is the reason our composition based SVM models discriminate toxic and non-toxic peptides with high precession [25], [30]. Dipeptide is another important feature and has been found to perform better than composition-based model in many previous studies. In this study also dipeptide-based model perform better than models based on simple amino acid composition in predicting toxicity of peptides. In order to incorporate information regarding composition as well as order of amino acid, we have generated binary profiles and developed SVM models using these profiles as input features. Unfortunately, our SVM models based on binary profiles could not perform better than the composition-based models. In two sequence logos, Cys is the only predominant residue present at most of the positions, and this could be the most probable reason of the poor performance of binary profiles-based SVM model.

In this study, all datasets used for developing models are unbalanced, where number of non-toxic peptides is many folds than the toxic peptides (nearly two times in case of main dataset and seven times in case of alternate dataset). Thus we also developed models on balanced datasets. To develop main balanced dataset, we randomly picked up equal number of (1805) non-toxic peptides. We evaluated the performance of dipeptide-based model on this dataset using cross validation technique and achieved accuracy of 93.88% with 0.88 MCC. The performances of our models on balanced as well as on unbalanced datasets were nearly the same.

In addition, we also developed motif-based method using MEME/MAST [27] module. We developed this model considering that few patterns/motifs might be associated with toxicity of peptides. Therefore, first, various motifs were fished out from toxic peptides using MEME and then MAST module was used to scan these motifs in peptides. This approach has been used successfully in the past [25] and in the present study also, the motif-based model performed reasonably well. Finally, a hybrid model using both dipeptide composition and motif information was developed to improve the performance of the model. Motif information has further improved the performance of the hybrid model. However, one of the limitations of this hybrid model is that motif extraction and searching was not carried out by five-fold cross validation rather than motifs were extracted from all toxic peptides. In order to assist users, we have developed a user-friendly web server, ToxinPred, based on the best models (dipeptide and hybrid).

## Supporting Information

File S1 **File containing all supporting information figures and tables.** Figure S1: Sequence logos of (A) first ten residues of N-terminus and (B) last ten residues of C-terminus of toxic peptides (alternate dataset), where size of residue is proportional to its propensity. Figure S2: Maximum and minimum scoring residues at every position as observed in quantitative matrix (alternate dataset). Table S1: Performance of whole amino acid and dipeptide composition-based SVM model developed on alternate dataset. Table S2: Performance of Binary profile-based models developed on alternate dataset. Table S3: Performance of motif based prediction (on alternate dataset). Table S4: Performance of hybrid model developed on alternate dataset.
(DOC)
