# Supplementary material for: In Silico Approach for Predicting Toxicity of Peptides and Proteins
Source: PLoS One. 2013 Sep 13;8(9):e73957. doi: 10.1371/journal.pone.0073957 (PMC3772798; doi:10.1371/journal.pone.0073957)
Supplement: File S1 — File containing all supporting information figures and tables. Figure S1: Sequence logos of (A) first ten residues of N-terminus and (B) last ten residues of C-terminus of toxic peptides (alternate dataset), where size of residue is proportional to its propensity. Figure S2: Maximum and minimum scoring residues at every position as observed in quantitative matrix (alternate dataset). Table S1: Performance of whole amino acid and dipeptide composition-based SVM model developed on alternate dataset. Table S2: Performance of Binary profile-based models developed on alternate dataset. Table S3: Performance of motif based prediction (on alternate dataset). Table S4: Performance of hybrid model developed on alternate dataset. (DOC) [file pone.0073957.s001.doc]

Supplementary Information

*In Silico* Approach for Predicting Toxicity of Peptides and Proteins

Sudheer Gupta^1#^, Pallavi Kappor^1#^, Kumardeep Chaudhary^1#^, Ankur Gautam^1#^, Rahul Kumar^1^, Open Source Drug Discovery^2^, G.P.S. Raghava^1*^

**Figure S1.** Sequence logos of (A) first ten residues of N-terminus and (B) last ten residues of C-terminus of toxic peptides (alternate dataset), where size of residue is proportional to its propensity.

**
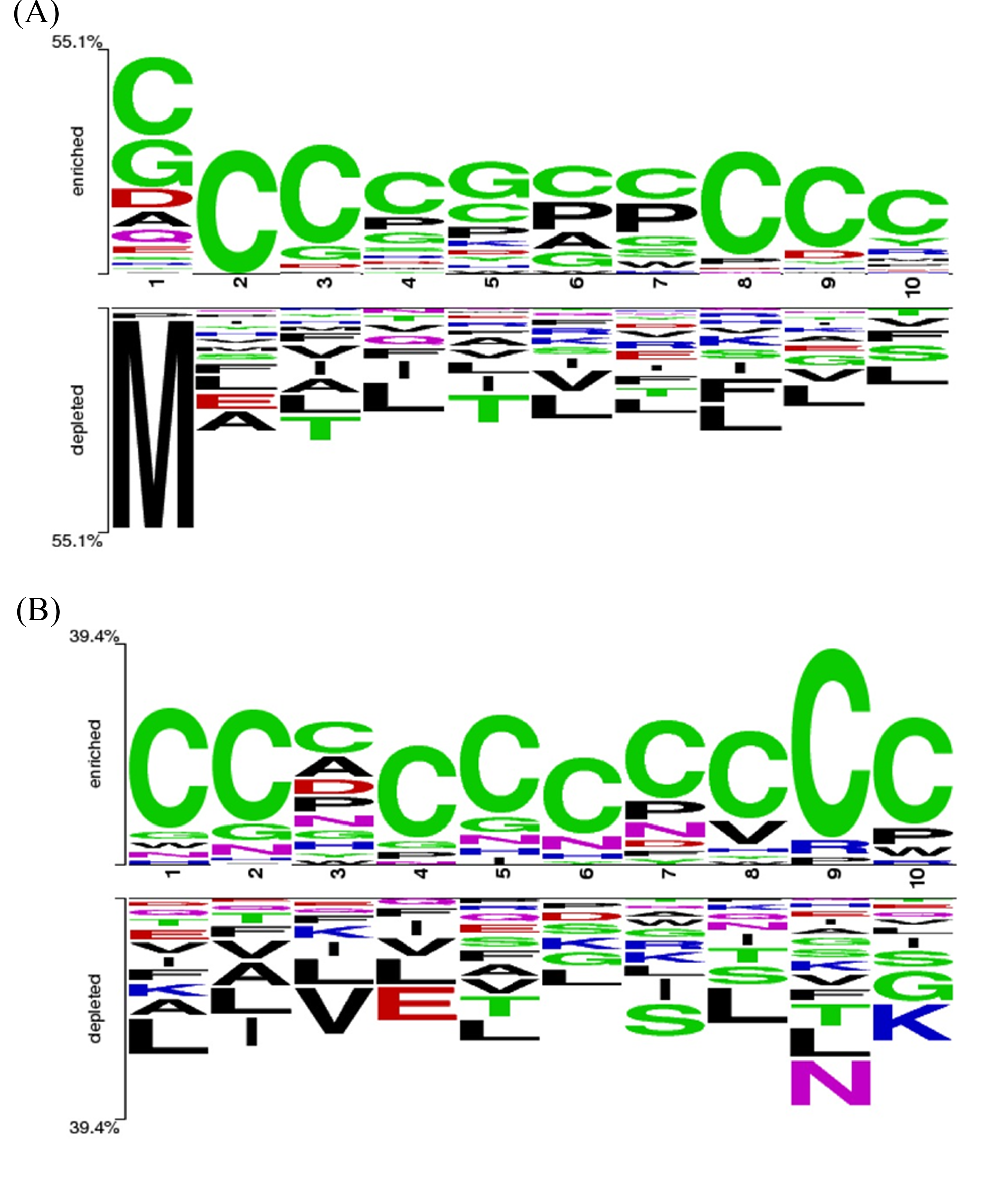
**

**Figure S2.** Maximum and minimum scoring residues at every position as observed in quantitative matrix (alternate dataset).

**
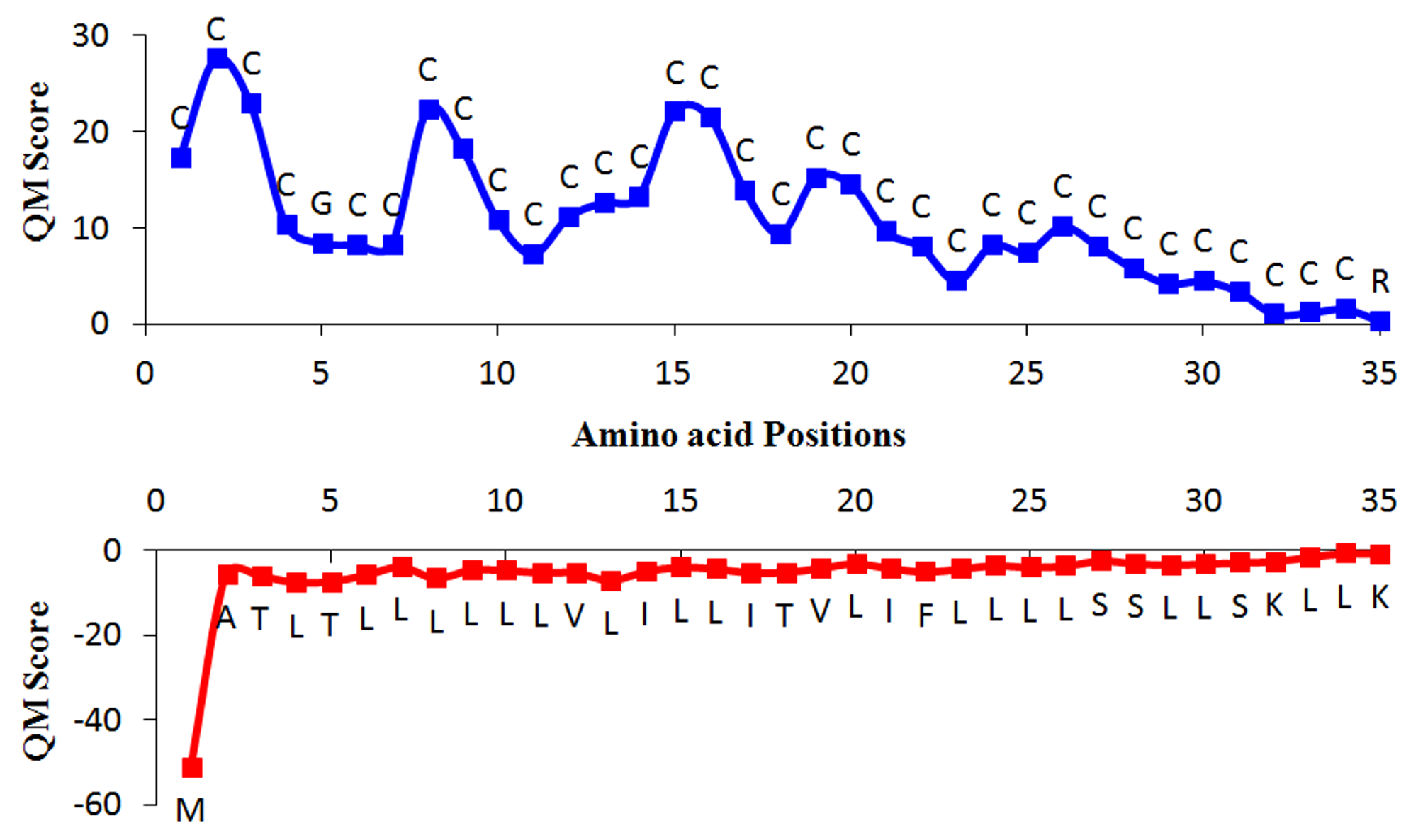
**

**Table S1:** Performance of whole amino acid and dipeptide composition-based SVM model developed on alternate dataset.

| **Features** | **Threshold** | **Sensitivity** | **Specificity** | **Accuracy** | **MCC** | **AUC** |
| --- | --- | --- | --- | --- | --- | --- |
| **AAC** | -0.6 | 95.62 | 96.86 | 96.70 | 0.86 | 0.99 |
| **C5AAC** | -0.9 | 82.81 | 83.30 | 83.24 | 0.51 | 0.88 |
| **C10AAC** | -0.7 | 88.94 | 88.56 | 88.61 | 0.64 | 0.95 |
| **N5AAC** | -0.8 | 83.35 | 85.92 | 85.59 | 0.55 | 0.91 |
| **N10AAC** | -0.7 | 86.02 | 84.03 | 84.29 | 0.55 | 0.91 |
| **DPC** | **0** | **91.80** | **99.63** | **98.64** | **0.94** | **0.99** |

AAC, amino acid composition; DPC, dipeptide composition; C5AAC, amino acid composition of last five C-terminal residues; C10AAC, amino acid composition of last ten C-terminal residues; N5AAC, amino acid composition of first five N-terminal residues; N10AAC, amino acid composition of first ten N-terminal residues; MCC, Matthew’s correlation coefficient; AUC, area under the curve.

**Table S2.** Performance of Binary profile-based models developed on alternate dataset.

| **Feature** | **Threshold** | **Sensitivity** | **Specificity** | **Accuracy** | **MCC** | **AUC** |
| --- | --- | --- | --- | --- | --- | --- |
| CT5 | -0.6 | 92.42 | 89.54 | 89.94 | 0.68 | 0.96 |
| CT10 | -0.9 | 85.64 | 90.91 | 90.23 | 0.65 | 0.95 |
| NT5 | -0.8 | 93.01 | 92.12 | 92.24 | 0.74 | 0.98 |
| NT10 | -0.7 | 86.02 | 84.03 | 84.29 | 0.55 | 0.91 |

MCC, Matthew’s correlation coefficient; AUC, area under the curve.

**Table S3. Performance of motif based prediction (on alternate dataset).**

| **E-value** | **PCP** | **%Coverage** |
| --- | --- | --- |
| **10** | 16.34 | 93.54 |
| **1** | 22.20 | 90.08 |
| **0.1** | 31.43 | 86.28 |
| **0.01** | 45.13 | 82.31 |
| **1E-02** | 61.73 | 78.29 |
| **1E-04** | 75.91 | 74.83 |
| **1E-05** | 85.89 | 71.77 |
| **1E-06** | 92.40 | 68.25 |
| **1E-07** | 96.59 | 64.17 |

PCP, probability of correct prediction

**Table S4.** Performance of hybrid model developed on alternate dataset.

| **E-value** | **Sensitivity** | **Specificity** | **Accuracy** | **MCC** | **AUC** |
| --- | --- | --- | --- | --- | --- |
| **10** | 99.67 | 97.88 | 98.63 | 0.97 | 0.99 |
| **1** | 99.39 | 97.88 | 98.51 | 0.97 | 0.99 |
| **0.1** | 99.00 | 97.88 | 98.35 | 0.97 | 0.99 |
| **0.01** | 98.56 | 97.88 | 98.16 | 0.96 | 0.99 |
| **0.001** | 98.17 | 97.88 | 98.00 | 0.96 | 0.99 |
| **0.0001** | 98.06 | 97.88 | 97.96 | 0.96 | 0.99 |
| **0.00001** | 97.89 | 97.88 | 97.89 | 0.96 | 0.99 |
| **0.000001** | 97.78 | 97.88 | 97.84 | 0.96 | 0.96 |
| **0.0000001** | 97.51 | 97.88 | 97.72 | 0.95 | 0.99 |

MCC, Matthew’s correlation coefficient; AUC, area under the curve.
